# Pharmacokinetic and exposure–response analysis of pertuzumab in patients with HER2-positive metastatic gastric or gastroesophageal junction cancer

**DOI:** 10.1007/s00280-019-03871-w

**Published:** 2019-06-10

**Authors:** Whitney P. Kirschbrown, Bei Wang, Ihsan Nijem, Atsushi Ohtsu, Paulo M. Hoff, Manish A. Shah, Lin Shen, Yoon-Koo Kang, Maria Alsina, Sandhya Girish, Amit Garg

**Affiliations:** 1 Clinical Pharmacology, Genentech Research and Early Development, DNA Way, MS463a, South San Francisco, CA 94080 USA; 2 Department of Gastrointestinal Oncology, National Cancer Center Hospital East, Kashiwa, Japan; 3 Instituto do Câncer de São Paulo, Faculdade de Medicina da Universidade de São Paulo, São Paulo, Brazil; 4 Medical Oncology/Solid Tumor Program, Sandra and Edward Meyer Cancer Center, Weill Cornell Medical College, New York, NY USA; 5 Key Laboratory of Carcinogenesis and Translational Research (Ministry of Education/Beijing), Department of Gastrointestinal Oncology, Peking University Cancer Hospital and Institute, Beijing, China; 6 Department of Oncology, Asan Medical Center, University of Ulsan College of Medicine, Seoul, Korea; 7 Vall d’Hebron University Hospital and Institute of Oncology (VHIO), Universitat Autònoma de Barcelona, Barcelona, Spain

**Keywords:** Pertuzumab, Trastuzumab, Pharmacokinetics, HER2-positive, Metastatic gastroesophageal junction, Metastatic gastric cancer

## Abstract

**Purpose:**

To characterize the pharmacokinetics (PK) of pertuzumab and trastuzumab in patients with HER2-positive metastatic gastric or gastroesophageal junction cancer in the randomized, double-blind, phase III JACOB study (NCT01774786), and to evaluate the appropriateness of the pertuzumab regimen in these patients.

**Methods:**

Patients received 840 mg intravenous pertuzumab or placebo plus trastuzumab q3w and chemotherapy. Pertuzumab and trastuzumab were administered until disease progression or unacceptable toxicity. Chemotherapy was administered for up to six cycles or disease progression or unacceptable toxicity. Serum concentrations of pertuzumab and trastuzumab were measured. Pertuzumab PK was characterized across treatment cycles. The impact of anti-drug antibodies (ADAs) on pertuzumab PK and the impact of pertuzumab on trastuzumab PK were assessed. An exploratory exposure–efficacy analysis was also conducted.

**Results:**

In total, 374 patients in the pertuzumab arm had evaluable PK data. The mean observed pertuzumab steady-state serum trough (minimum) concentration (*C*_min,ss_) ± standard deviation was 114 ± 51.8 μg/mL. The target pertuzumab *C*_min,ss_ of ≥ 20 μg/mL was reached in 99.3% of patients at Cycle 5 (steady state) and beyond. Greater than 90% of patients were above the PK target right after the first pertuzumab dose. There was no apparent impact of ADAs on pertuzumab PK nor of pertuzumab on trastuzumab PK. There were no differences in overall survival across Cycle 1 pertuzumab (*C*_min_) or Cycle 5 pertuzumab (*C*_min,ss_) exposure quartiles.

**Conclusions:**

Pertuzumab exposure in JACOB was consistent with prior studies in advanced gastric cancer and breast cancer. The 840 mg q3w dose allowed the majority of patients in JACOB to achieve target pertuzumab concentrations and appears to be an appropriate dose selection.

**Electronic supplementary material:**

The online version of this article (10.1007/s00280-019-03871-w) contains supplementary material, which is available to authorized users.

## Introduction

Pertuzumab (PERJETA^®^, F. Hoffmann-La Roche Ltd, Basel, Switzerland) is a monoclonal antibody (mAb) that inhibits human epidermal growth factor receptor 2 (HER2) dimerization with other HER family receptors, thereby inhibiting mitogen-activated protein kinase and phosphoinositide 3-kinase signaling pathways and promoting cell-growth arrest and apoptosis [1, 2].

Adding pertuzumab to trastuzumab (Herceptin^®^, F. Hoffmann-La Roche Ltd) may provide a more comprehensive HER2 pathway blockade (vs. trastuzumab alone) due to the complementary modes of action of the two drugs [3]. The addition of pertuzumab to trastuzumab has been shown to improve survival outcomes in patients with HER2-positive early breast cancer (EBC) and metastatic breast cancer (MBC) [4–6]. Although there are differences in tumor biology between HER2-positive breast cancer and HER2-positive gastric cancer (GC), it was hypothesized that the dual HER2-targeted regimen could also improve survival outcomes in patients with HER2-positive advanced GC (AGC) [7].

The pharmacokinetics (PK) and safety of pertuzumab plus trastuzumab and chemotherapy in patients with HER2-positive AGC was initially evaluated in the phase IIa dose-finding JOSHUA study (NCT01461057) [7]. This study showed that the combination of pertuzumab, trastuzumab, and chemotherapy was well tolerated in these patients, with an adverse event (AE) profile similar to that seen in the ToGA trial (trastuzumab and chemotherapy vs. chemotherapy alone; NCT01041404) [8]. The PK analysis of JOSHUA found that mean serum trough levels at day 43 were 37% lower in patients with HER2-positive AGC, compared with patients with HER2-positive MBC when the same dose was administered in both indications [7, 9]. PK analyses also showed that the new dosing regimen of 840 mg every 3 weeks (q3w) resulted in higher pertuzumab concentrations and increased the probability of patients achieving a target serum trough concentration (*C*_min_) of pertuzumab similar to those observed in HER2-positive EBC and MBC with the previously established 840 mg loading dose followed by 420 mg q3w approved in those indications, without compromising the overall safety profile of the regimen [7]. The pertuzumab serum concentration of > 20 μg/mL was established as the target efficacious exposure (*C*_min_) based on non-clinical efficacy models [10].

JACOB (NCT01774786) was a prospective, randomized, multicenter, multinational, double-blind, placebo-controlled, phase III trial that evaluated the efficacy and safety of the new pertuzumab dosing regimen of 840 mg q3w administered intravenously (IV) plus trastuzumab and chemotherapy (pertuzumab arm), compared with placebo plus trastuzumab and chemotherapy (placebo arm) as first-line therapy in patients with previously untreated HER2-positive metastatic GC or gastroesophageal junction cancer (MGC/GEJC) [11]. The JACOB study did not meet its primary endpoint of a statistically significant improvement in overall survival [OS; hazard ratio 0.84, 95% confidence interval (CI) 0.71–1.00, stratified log-rank *p* = 0.0565].

Here, we present data from the PK analysis of pertuzumab and trastuzumab in patients with MGC/GEJC in the JACOB study. A secondary objective of the JACOB study was to assess the pharmacokinetics of pertuzumab. Exploratory objectives included an assessment of PK drug–drug interactions (DDIs), pharmacokinetics of trastuzumab, the impact of anti-drug antibodies (ADAs) on PK, efficacy, and safety, effect of shed HER2 extracellular domain (ECD) on PK, and evaluation of the exposure–efficacy relationship, conducted to evaluate the appropriateness of the 840 mg q3w pertuzumab regimen.

## Methods

### Patients and study design

JACOB was a randomized, multicenter, multinational, double-blind, placebo-controlled, phase III trial evaluating the efficacy and safety of pertuzumab vs. placebo in combination with trastuzumab and chemotherapy. The JACOB study design has been described previously [11]. In brief, patients aged ≥ 18 years with HER2-positive MGC/GEJC, measurable or evaluable non-measurable disease at baseline, Eastern Cooperative Oncology Group performance status of 0 or 1, and baseline left ventricular ejection fraction ≥ 55% were eligible; patients who had received the previous therapy with an HER2-targeted drug or previous systemic chemotherapy for metastatic disease were excluded.

Enrolled patients were randomized 1:1 to receive pertuzumab or placebo (840 mg pertuzumab or placebo given IV q3w) plus trastuzumab (IV; 8 mg/kg loading dose followed by 6 mg/kg maintenance dose, q3w) and chemotherapy (oral capecitabine: 1000 mg/m^2^, taken twice daily on days 1–15, q3w, or 5-fluorouracil: 800 mg/m^2^/24 h IV by continuous infusion for 120 h on days 1–5, q3w; plus cisplatin: 80 mg/m^2^ IV on day 1 only, q3w). Randomization was stratified by geographic region (Japan vs. North America/Western Europe/Australia vs. Asia [excluding Japan] vs. South America/Eastern Europe), prior gastrectomy, and HER2 immunohistochemistry. Pertuzumab (or placebo) and trastuzumab were given until disease progression or unacceptable toxicity. Chemotherapy was given for six treatment cycles and only discontinued during or before Cycle 6 for progressive disease or unacceptable toxicity.

The JACOB study was conducted in accordance with the International Conference on Harmonisation E6 guideline for Good Clinical Practice (ICH–GCP E6) and the principles of the Declaration of Helsinki, or the laws and regulations of the country in which the research was done; whichever provided the greater protection for the individual.

### PK, anti-drug antibodies, and HER2 extracellular domain sampling

PK samples were taken from all patients in the JACOB study for the PK analysis of pertuzumab and trastuzumab. Serum samples were collected pre-dose at Cycles 1, 2, 3, 4, 6, and 8 and post-infusion at Cycles 1, 2, 4, and 8. Cycle 8 serum samples were collected when patients were on biologic therapy (i.e., pertuzumab and/or trastuzumab) alone and the last chemotherapy administration was ≥ 6 weeks ago. Two additional serum samples were collected at post-treatment monitoring visits at 28 (± 7) days and 60‒90 days after last study treatment administration (post-treatment monitoring visits 1 and 2, respectively) to support interpretation of anti-drug antibody (ADA) with minimized potential for drug interference in the ADA assay. These samples were analyzed for pertuzumab (pertuzumab arm only) and trastuzumab (pertuzumab and placebo arms) concentrations by PPD^®^ Laboratories, LLC (Richmond, VA, USA) using validated assays.

Based on historical data in EBC and MBC, pertuzumab steady state should be reached at day 43, following one loading and one maintenance dose [1, 10]. Pertuzumab PK characterization in this study was performed using observed data only, not model predicted, which could account for time-dependent clearance. Therefore, the latest cycle of observed data, where most patients remained on study (Cycle 5), was used to designate steady state.

Prior pertuzumab population PK (popPK) analyses did not indicate that geographic region or race were covariates on clearance or volume; however, geographic region was one of the stratification factors of the JACOB study, as different regions have different screening and early detection practices, which could influence efficacy. Therefore, ensuring no pertuzumab exposure differences in this large global trial was warranted.

Serum samples to test for the presence of ADAs against pertuzumab were collected from all patients pre-dose at Cycles 1, 3, and 6 and at post-treatment monitoring visits 1 and 2. The samples were analyzed for ADAs against pertuzumab (pertuzumab arm only) by PPD^®^ Laboratories, LLC using a validated immunoassay.

Separate blood samples were obtained for the assessment of serum concentration of shed HER2 extracellular domain (ECD) at pre-dose at Cycles 1, 3, and 6, and during post-treatment monitoring visit 1. The samples were analyzed for shed HER2 ECD by Covance Laboratories (Indianapolis, IN, USA) using a validated immunoassay.

### Bioanalytical methods

Serum concentrations of pertuzumab were determined by a validated enzyme-linked immunosorbent assay (ELISA) with a minimum quantifiable concentration of 150 ng/mL [12]. The ELISA assay showed acceptable accuracy (% difference) and inter-assay percent coefficient of variation (% CV) with ranges of − 8.75 to 3.84% and 3.89–15.3%, respectively.

Serum concentrations of trastuzumab were determined by a validated high-performance liquid chromatography assay with tandem mass spectrometry detection (minimum quantifiable concentration of 100 ng/mL) [13]. This assay showed acceptable accuracy (% difference) and % CV with ranges of − 8.08 to − 1.47% and 3.07 to 8.44%, respectively.

A validated ELISA was used to detect and confirm the presence of ADAs to pertuzumab in human serum. This assay used two conjugated reagents to capture ADAs directed against pertuzumab: biotin-conjugated pertuzumab and digoxin-conjugated pertuzumab. Bound ADAs to pertuzumab were then detected with a mouse anti-digoxin antibody conjugated with horseradish peroxidase. Using an anti-idiotypic mAb directed against pertuzumab as a positive control, the relative sensitivity was determined to be 3.59 ng/mL in the absence of pertuzumab. In the presence of 200 μg/mL pertuzumab, the assay can detect 500 ng/mL of the anti-idiotypic mAb control.

Serum concentrations of HER2 ECD were measured using the commercially available ADVIA Centaur^®^ Serum HER-2/neu Assay (Siemens Healthcare Diagnostics Inc., Deerfield, IL, USA), a fully automated two-site sandwich immunoassay using direct chemiluminescent technology. The immunoassay has a minimum detectable concentration of 0.5 ng/mL; the assay showed acceptable accuracy (% recovery) and % CV with ranges of 88.7–100.9 and 3.2–5.7%, respectively.

### Data handling

All patients treated who had at least one documented pertuzumab or trastuzumab administration and at least one corresponding measurable concentration of pertuzumab or trastuzumab were included in the PK analysis, unless there were major protocol deviations or information that may have interfered with PK evaluation (i.e., labeling error, technical failure in sample analysis). Records were excluded if the time of drug administration or sample collection was missing. No imputation of PK values was performed. Observations with missing PK or time values, or those below the lower limit of quantification, were omitted from the analysis.

### PK assessments

Peak (maximum) serum concentrations *C*_max_ (post-dose) and *C*_min_ (pre-dose) from prespecified collection timepoints were summarized using descriptive statistics and graphical assessment. *C*_min_ refers to the concentration at the end of a dosing interval, and therefore, the pre-dose PK sample of a given cycle refers to the previous cycle’s *C*_min_ (i.e., pre-dose Cycle 6 is the Cycle 5 *C*_min_). To reduce burden for patients and healthcare providers, sparse PK sampling was used, where only pre-dose and a few post-dose samples were collected. Steady-state concentrations of pertuzumab (*C*_max,ss_ and *C*_min,ss_) from JACOB were compared across different geographic regions (a prespecified stratification factor in the study): Japan, North America/Western Europe/Australia, Asia (excluding Japan), and South America/Eastern Europe. Steady-state concentrations of pertuzumab from JACOB were compared with equivalent data obtained in the previous studies of women with MBC (CLEOPATRA, NCT00567190) [9] and patients with AGC (JOSHUA) [7]. Similarly, steady-state concentrations of trastuzumab from JACOB were compared with equivalent data obtained in the previous AGC studies (JOSHUA and ToGA) [7, 14].

### Analysis of DDIs

Potential effects of pertuzumab on the steady-state PK of trastuzumab were assessed by comparing the arithmetic means of serum trastuzumab concentrations at pre-dose Cycle 6 (Cycle 5 *C*_min,ss_) and post-dose (*C*_max,ss_) in Cycle 4 between the pertuzumab and placebo arms. In addition, the 90% CI for the ratio of the geometric means were constructed. Potential effects of chemotherapy on pertuzumab or trastuzumab PK were assessed by comparing the arithmetic means of *C*_min_ in Cycle 5 (with chemotherapy) and Cycle 7 (without chemotherapy). If the 90% CI of the ratio of arithmetic means was contained within 80–125%, no apparent DDI was concluded.

### Exploratory exposure–efficacy analyses

Observed individual pertuzumab exposures at Cycle 1 and at steady state from patients in the pertuzumab arm were used in the exploratory exposure–efficacy analysis. The efficacy endpoint in the analysis was the primary study endpoint, OS. Patients who had not had an event at the time of data analysis were censored at the date they were last known to be event free. The primary exposure metrics used in the exposure–efficacy analysis were individual-observed Cycle 1 *C*_min_ and Cycle 5 *C*_min,ss_. Individuals who died prior to Cycle 6 were not included in the analysis. Kaplan–Meier curves were generated to determine survival probability within Cycle 1 and Cycle 5 *C*_min_ quartiles. A log-rank test determined whether statistically significant survival differences existed among *C*_min_ quartiles.

### Assessment of ADAs on PK, safety, and efficacy

Incidence of ADAs to pertuzumab was measured and the impact of ADAs on pertuzumab PK, safety, and efficacy was assessed.

### Assessment of HER2 ECD concentrations

Serum concentrations of HER2 ECD were measured in both treatment arms at baseline. The effect of shed HER2 ECD on pertuzumab PK was assessed.

## Results

### Study population and demographics

In JACOB, 780 patients were randomized to receive pertuzumab plus trastuzumab and chemotherapy (*n* = 388) or placebo plus trastuzumab and chemotherapy (*n* = 392). Of these, 374 patients in the pertuzumab arm and 375 patients in the placebo arm had evaluable PK data and were included in this analysis. Patient demographics and disease characteristics at baseline in the intention-to-treat population are provided in Online Resource 1 [11].

### PK analyses

Summary statistics of pertuzumab PK in patients treated with pertuzumab are presented in Table 1. Following the first IV administration of pertuzumab 840 mg, the mean observed *C*_max_ ± standard deviation (SD) for pertuzumab was 258 ± 90.3 μg/mL. The mean observed *C*_min,ss_ ± SD of pertuzumab following five subsequent pertuzumab doses at 840 mg q3w at Cycle 5 was 114 ± 51.8 μg/mL (Table 1). The target *C*_min,ss_ of ≥ 20 μg/mL was reached in 99.3% of patients at Cycle 5 *C*_min,ss_ and beyond. In addition, > 90% of patients were above the PK target right after the first pertuzumab dose.

**Table 1:** Summary of pharmacokinetic parameters

Drug substance (treatment arm)	*C*_max_ Cycle 1 (μg/mL)	*C*_min_ Cycle 1 (μg/mL)	*C*_min_ Cycle 3 (μg/mL)	*C*_max_ Cycle 4 (μg/mL)	*C*_min_ Cycle 5 (μg/mL)	*C*_min_ Cycle 7 (μg/mL)	*C*_max_ Cycle 8 (μg/mL)
Pertuzumab (pertuzumab arm)							
*n*	374	349	305	302	274	114	106
Mean (SD)	258 (90.3)	42.4 (24.8)	90.4 (42.4)	341(111)	114 (51.8)	142 (67.9)	371 (127)
Trastuzumab (pertuzumab arm)							
*n*	372	345	305	304	274	114	115
Mean (SD)	142 (86.8)	15.4 (11.3)	22.9 (12.7)	127 (50.9)	26.3 (14.8)	32.7 (15.0)	130 (50.8)
Trastuzumab (placebo arm)							
*n*	375	354	300	299	254	92	90
Mean (SD)	139 (58.6)	17.2 (15.4)	24.1 (19.0)	129 (58.1)	29.8 (21.9)	37.4 (20.3)	147 (90.2)

*C*_*max*_ peak (maximum) serum concentration, *C*_*min*_ serum trough (minimum) concentration, *SD* standard deviation

Summary statistics of trastuzumab PK in patients treated with trastuzumab (pertuzumab and placebo arms) are presented in Table 1. Following the loading dose IV administration of trastuzumab at 8 mg/kg, the mean observed *C*_max_ ± SD of trastuzumab was 142 ± 86.8 μg/mL in the pertuzumab arm and 139 ± 58.6 μg/mL in the placebo arm (Table 1). The mean observed *C*_min,ss_ of trastuzumab following five subsequent trastuzumab maintenance doses of 6 mg/kg q3w in Cycle 5 was 26.3 ± 14.8 μg/mL in the pertuzumab arm and 29.8 ± 21.9 μg/mL in the placebo arm (Table 1).

### Comparison of pertuzumab and trastuzumab PK data from JACOB with the previous studies in MBC and AGC, and across geographic regions

**Fig. 1:**
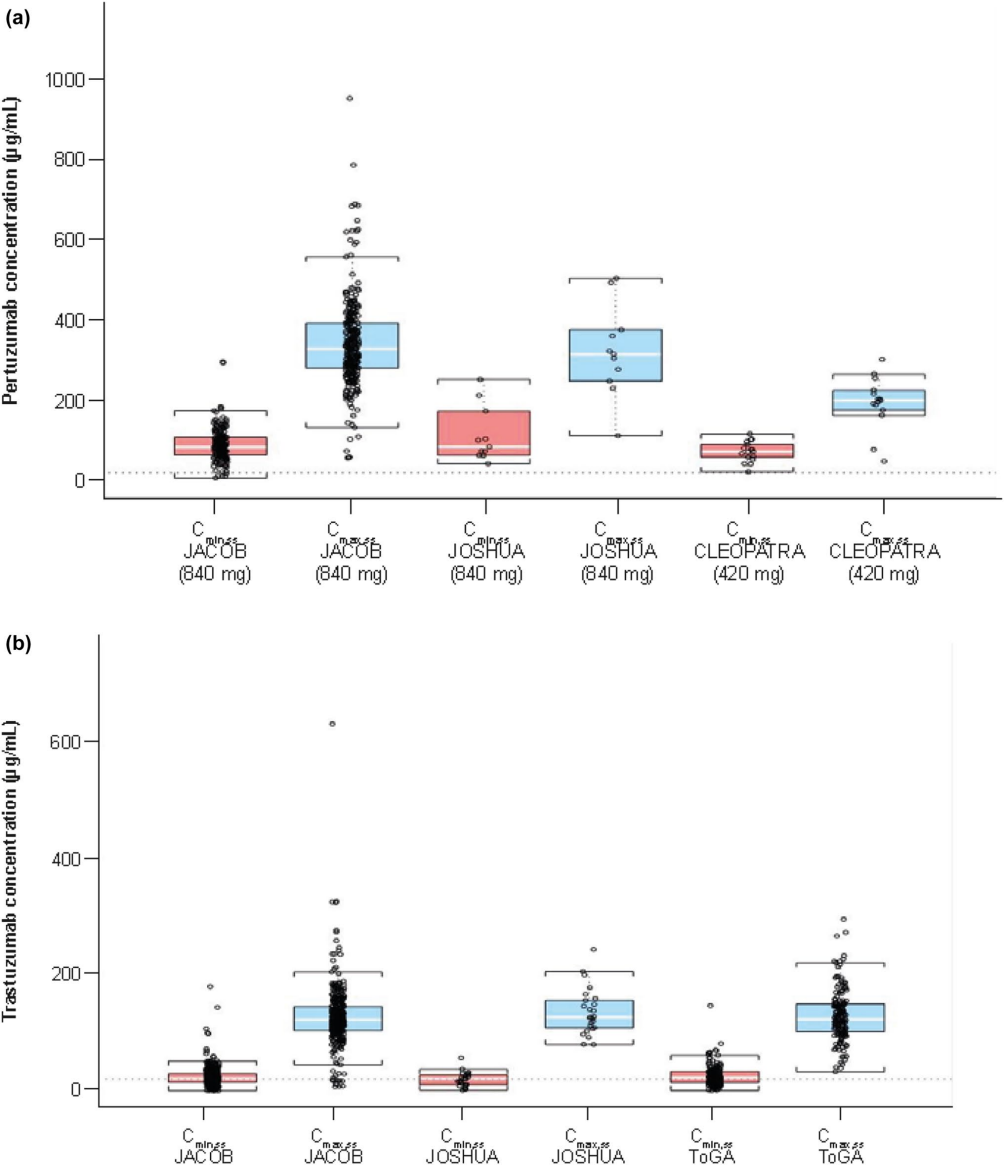
Cross-study comparison of PK for **a** pertuzumab [7, 9, 11] and **b** trastuzumab [7, 11, 14]. *C*_*max,ss*_ steady-state peak (maximum) serum concentration, *C*_*min,ss*_ steady-state serum trough (minimum) concentration, *PK* pharmacokinetic. Cycle numbers for *C*_min,ss_ and *C*_max,ss_ vary between studies. *C*_min,ss_: JACOB, Cycle 3 pre-dose; JOSHUA, Cycle 4 pre-dose; CLEOPATRA, Cycle 9 pre-dose; ToGA, Cycle 9 pre-dose. *C*_max,ss_: JACOB, Cycle 4 post-dose; JOSHUA, Cycle 4 post-dose; CLEOPATRA, Cycle 9 post-dose; ToGA, Cycle 5 post-dose. Red bars = *C*_min_; blue bars = C_max_; lower and upper ends of each box plot = 25th and 75th percentile exposure value; horizontal white line = median per group; points = individual PK data. Brackets extending from the ends of the box are drawn to the nearest value, not beyond 1.5 times the interquartile range

Pertuzumab *C*_min,ss_ from JACOB was comparable to the previous studies in MBC (CLEOPATRA; 840 mg loading dose followed by 420 mg maintenance dose q3w) [9] and AGC (JOSHUA; 840 mg q3w arm) [7] (Fig. 1a). The higher pertuzumab dose in the JACOB study was reflected in higher *C*_max_ concentrations at steady state, compared with CLEOPATRA (mean *C*_max_ ± SD: 341 ± 111 vs. 196 ± 66.3) and consistent with *C*_max_ in JOSHUA (mean *C*_max_ ± SD [840 mg q3w arm]: 316 ± 134 μg/mL). Steady-state trastuzumab PK from JACOB was comparable to the previous studies in AGC (JOSHUA [840 mg q3w arm] [7] and ToGA [14]) (Fig. 1b).

*C*_min,ss_ for pertuzumab across the different geographic regions are shown in Online Resource 2. Pertuzumab exposure was comparable across geographic regions.

### DDIs

**Fig. 2:**
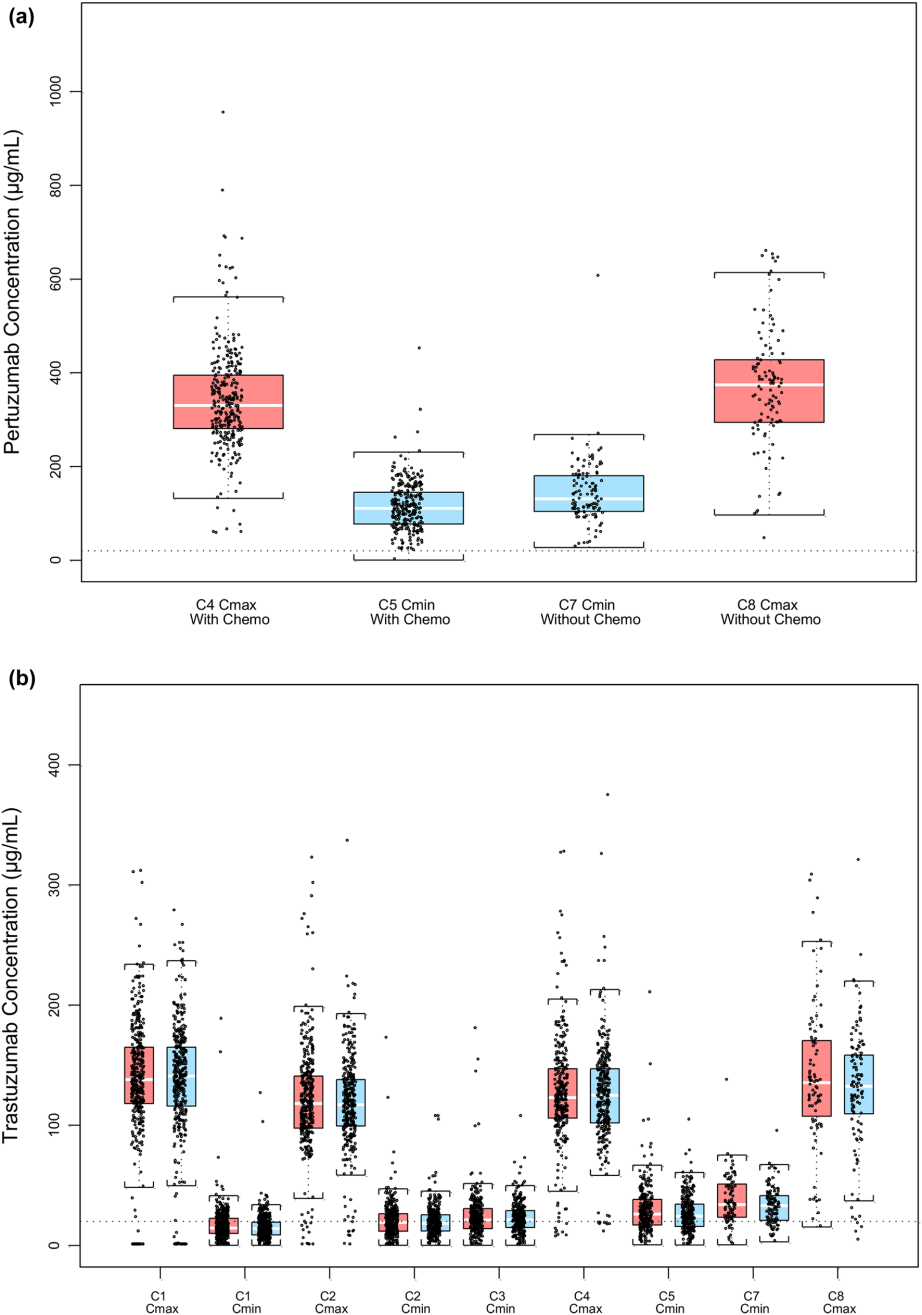
Drug–drug interaction assessment of the potential effects of **a** chemotherapy on pertuzumab exposure and **b** pertuzumab on trastuzumab exposure. *C* Cycle, *Chemo* chemotherapy, *C*_*max*_ peak (maximum) serum concentration, *C*_*min*_ serum trough (minimum) concentration, *PK* pharmacokinetic. Red bars = post-dose; blue bars = pre-dose; lower and upper ends of each box plot = 25th and 75th percentile exposure value; horizontal white line = median per group; points = individual PK data. Brackets extending from the ends of the box are drawn to the nearest value, not beyond 1.5-times the interquartile range

The ratio of arithmetic means of *C*_min,ss_ serum trastuzumab concentrations at Cycle 5 (pre-dose Cycle 6) in the pertuzumab versus placebo arm was 90.9% (90% CI 81.5–101.5). The ratio of arithmetic means of *C*_max,ss_ serum trastuzumab concentrations at Cycle 4 in the pertuzumab versus placebo arm was 101.5% (90% CI 94.8–108.8). Trastuzumab PK parameters were comparable between the pertuzumab and placebo arms, suggesting that adding pertuzumab-to-trastuzumab treatment did not alter the PK of trastuzumab (Fig. 2).

For patients in the pertuzumab arm who completed the last chemotherapy treatment in Cycle 6 and continued pertuzumab 840 mg treatment without chemotherapy thereafter, the Cycle 7 observed that mean *C*_min_ of pertuzumab was 142 ± 67.9 μg/mL. This was comparable to the pertuzumab PK exposure in Cycle 5 when patients were treated with pertuzumab in combination with trastuzumab and chemotherapy (Fig. 2a), indicating that chemotherapy had no apparent impact on pertuzumab PK.

For patients who completed the last chemotherapy treatment in Cycle 6 and continued 6 mg/kg trastuzumab without chemotherapy thereafter, the Cycle 7 observed mean *C*_min_ of trastuzumab was 32.7 ± 15.0 μg/mL in the pertuzumab arm and 37.4 ± 20.3 μg/mL in the placebo arm, which was comparable to the PK exposure in Cycle 5 when patients were treated with pertuzumab or placebo in combination with trastuzumab and chemotherapy (Fig. 2b), suggesting that chemotherapy had no impact on trastuzumab PK.

### Exposure–efficacy relationships

**Fig. 3:**
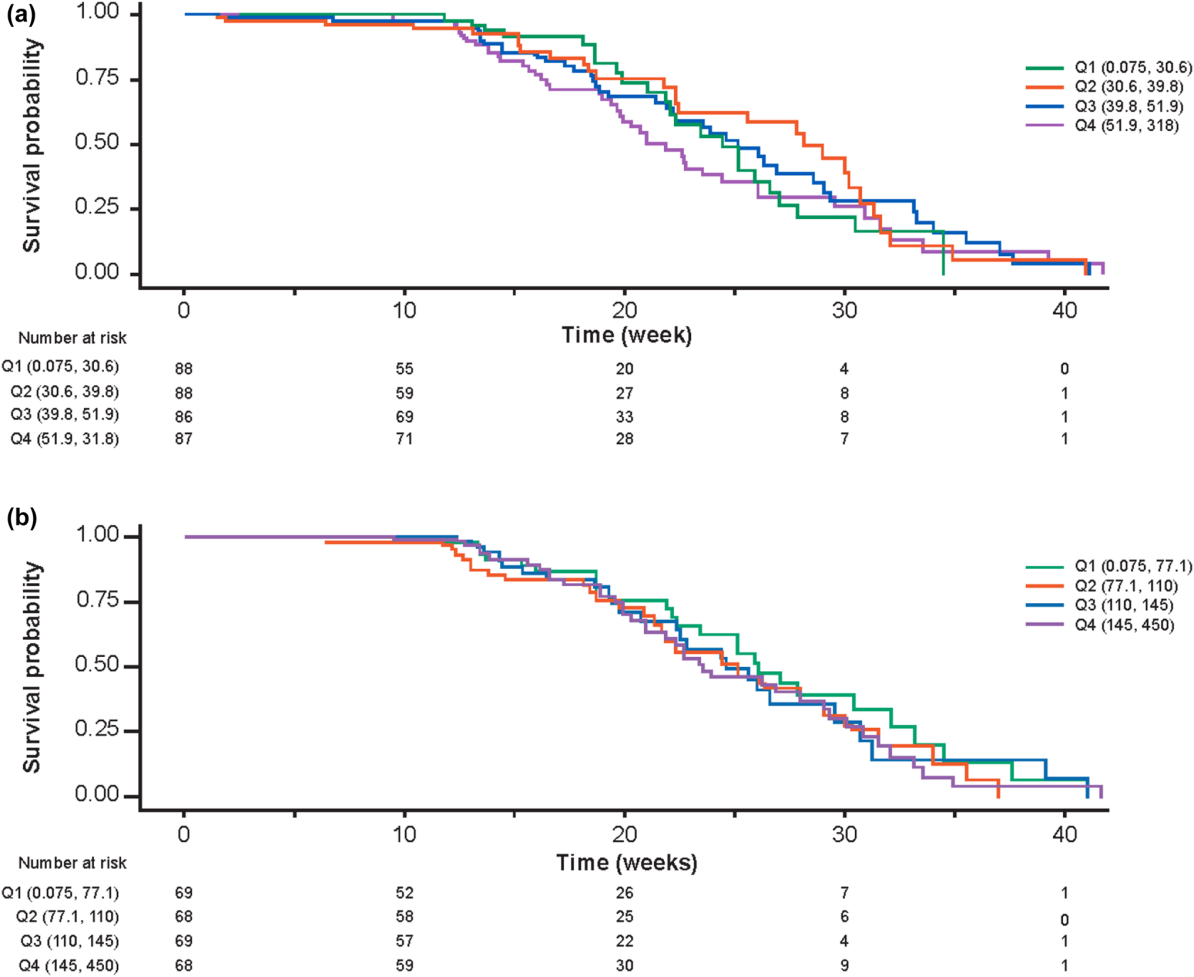
Kaplan–Meier plot of overall survival by pertuzumab exposure quartiles at **a** Cycle 1 (*C*_min_) and **b** Cycle 5 (*C*_min,ss_)

There were no statistically significant differences in OS across Cycle 1 pertuzumab *C*_min_ or Cycle 5 pertuzumab *C*_min,ss_ quartiles (Fig. 3). The median OS in weeks for Cycle 1 pertuzumab *C*_min_ quartiles were Q1: 43, Q2: 23, Q3: 28, and Q4: 40 (*n* = 349, *p* = 0.52) and the median OS in weeks for Cycle 5 pertuzumab *C*_min,ss_ quartiles were Q1: 26, Q2: 28, Q3: 26, and Q4: 38 (*n* = 274, *p* = 0.78).

### Immunogenicity

At baseline, 746 patients were evaluable for ADAs (pertuzumab arm, *n* = 376; placebo arm, *n* = 370); 21 patients in the pertuzumab arm and 14 patients in the placebo arm were ADA-positive at baseline (likely reflecting false positives or pre-existing ADAs). Of the 347 ADA-evaluable patients in the pertuzumab arm who had at least one post-dose sample available, only two were ADA-positive (0.6%). The pertuzumab *C*_min,ss_ for the two ADA-positive patients—135 ug/mL and 59.8 ug/mL, respectively—are within the range observed in the studied population.

### Effect of baseline shed HER2 ECD on pertuzumab PK

**Fig. 4:**
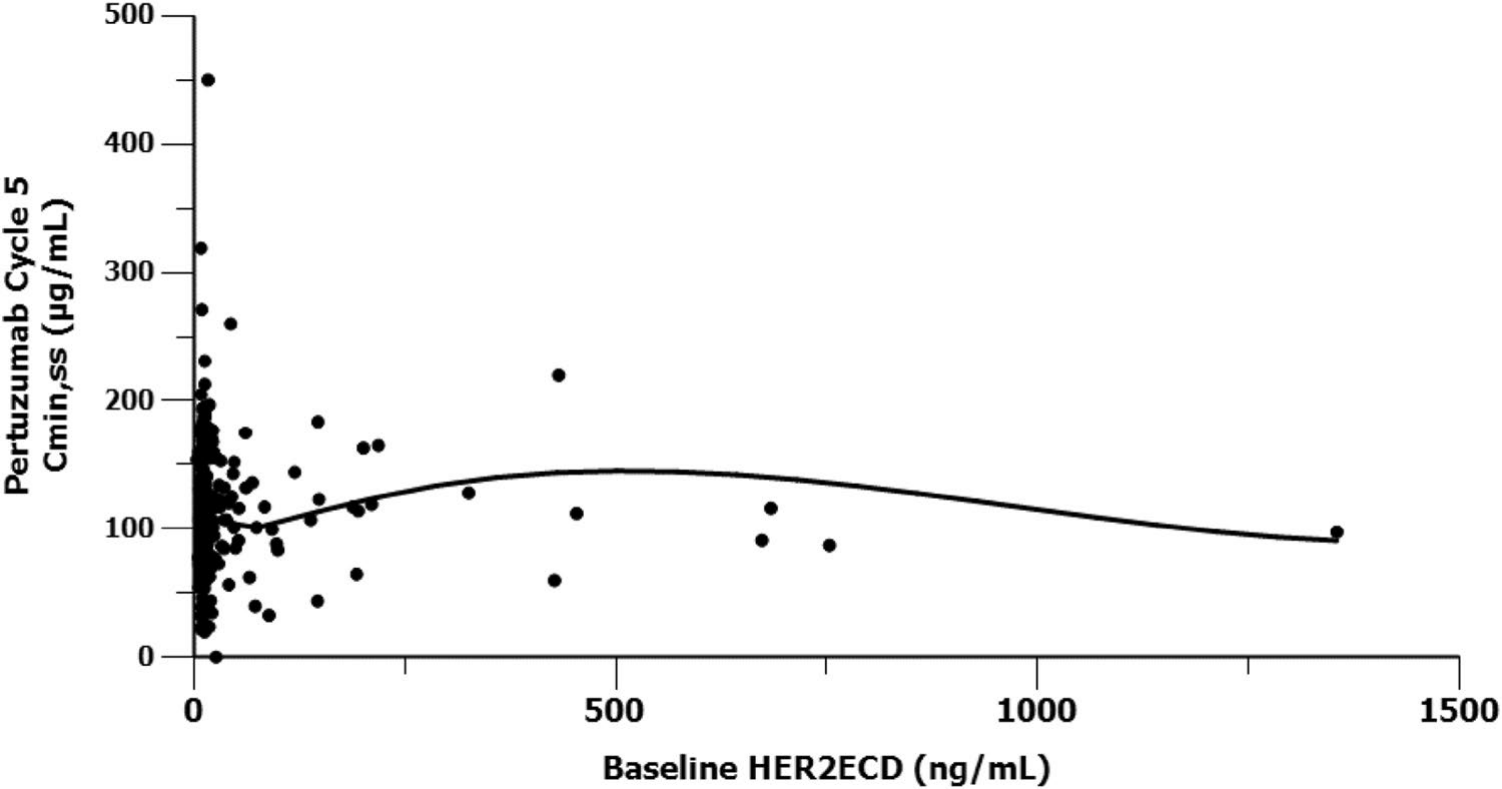
Pertuzumab steady-state exposure vs. HER2 ECD concentrations. *C*_*min*_ serum trough (minimum) concentration, *ECD* extracellular domain, *HER2* human epidermal growth factor receptor 2

There was considerable inter-patient variability in baseline HER2 ECD concentrations, as reflected by the broad ranges of individual values (3.5–2086.4 ng/mL) observed in both treatment arms of the study; however, most patients had baseline HER2 ECD concentrations below 100 ng/mL. In the pertuzumab arm, the mean ± SD and min–max baseline ECD concentrations were 42.1 ± 119 ng/mL and 3.5–1350 ng/mL, respectively (*n* = 361). Similar results were observed in the placebo treatment arm. No apparent relationship was seen between pertuzumab *C*_min,ss_ and baseline HER2 ECD concentrations (Fig. 4). HER2 ECD concentrations observed in JACOB were comparable to concentrations observed previously in breast cancer (F. Hoffmann-La Roche Ltd. Data on file).

## Discussion

The JACOB trial did not meet its primary endpoint of showing a statistically significant improvement in OS in patients who received pertuzumab in addition to trastuzumab and chemotherapy, compared to those who did not receive pertuzumab. However, there was utility in conducting a PK analysis to better understand the pertuzumab PK–pharmacodynamic relationship and to assess the appropriateness of the new pertuzumab dosing regimen selected for this indication. Collection of serum samples from all patients who participated in the JACOB study enabled this extensive characterization of the PK of pertuzumab in patients with HER2-positive MGC/GEJC.

The appropriateness of an 840 mg q3w dose was assessed in an exposure–efficacy analysis, which showed no statistically significant relationship between pertuzumab exposure quartiles and the probability of survival. Our findings, therefore, provide no evidence of additional efficacy with increasing pertuzumab exposure in this trial. Given the overall JACOB study outcome, which showed that OS was not significantly different between treatment and control (median OS 17.5 months [95% CI 16.2–19.3] in the pertuzumab arm and 14.2 months [95% CI 12.9–15.5] in the control arm; hazard ratio 0.84 [95% CI 0.71–1.00]; *p* = 0.57), the power to detect a difference between exposure quartiles in the treatment arm would be predictably very small.

In JACOB, 99.3% of patients with HER2-positive MGC/GEJC achieved target *C*_min,ss_, the target efficacious exposure based on the previous non-clinical efficacy models [10] when receiving the 840 mg q3w pertuzumab dose. In comparison, pertuzumab PK data from the registrational phase II NeoSphere study (pertuzumab, trastuzumab, and docetaxel in the neoadjuvant treatment of HER2-positive EBC; NCT00545688) showed that the 840 mg loading dose followed by 420 mg q3w pertuzumab regimen resulted in 94% of patients reaching the target *C*_min,ss_ of > 20 μg/mL [10]. The exposure–response analysis from NeoSphere also showed that there was no significant impact (*p* = 0.996) on the probability of the primary study endpoint (pathologic complete response in the breast) with an increase in pertuzumab serum concentration beyond 20 μg/mL [10]. The findings of this exposure–response analysis were also replicated in the phase III APHINITY study (pertuzumab, trastuzumab, and chemotherapy for the adjuvant treatment of HER2-positive EBC; NCT01358877) [15]. These two studies in patients with breast cancer support the selection of ≥ 20 µg/mL as a rational target serum trough exposure level of pertuzumab for therapeutic efficacy. Therefore, the 840 mg q3w dose, which enabled target pertuzumab concentrations to be achieved in the vast majority of patients with HER2-positive MGC/GEJC in the JACOB study, appears to be an appropriate selection from a PK/pharmacodynamic perspective. Furthermore, efficacious exposures of trastuzumab were also obtained in JACOB, as evidenced by a consistent  % of patients reaching the target trastuzumab *C*_min_ as in the pivotal trial ToGA [14]. In addition, in JACOB, trastuzumab *C*_min_ at Cycles 1 and 5 were similar in both the pertuzumab and placebo treatment arms.

Pertuzumab was well tolerated in the JACOB study and the safety profile was generally similar between the two treatment groups [11]. No new or unexpected safety events were reported, which is important given that the dose of 840 mg pertuzumab every 3 weeks was double the maintenance dose currently approved for breast cancer treatment [11]. Given the lack of significant improvements in efficacy outcomes and that pertuzumab was well tolerated at a higher dose, decreasing the pertuzumab dose was not justified as a viable treatment option, and therefore, no exposure–safety analyses were warranted.

No DDIs were found in this study, which is as expected based on prior data on pertuzumab and trastuzumab PK [10, 12] and the distinct clearance mechanisms between mAbs and small-molecule chemotherapy agents [16–18]. Pertuzumab and trastuzumab bind to distinct epitopes of HER2 simultaneously without steric hinderance, providing an additional rationale for why no DDI was expected [19].

There was no apparent impact of ADAs on pertuzumab PK in patients who were ADA-positive, and no apparent impact on safety as neither ADA-positive patient had any serious immune-related AEs nor other AEs suggestive of hypersensitivity. Although data were limited, there was no apparent impact on efficacy, as both patients were identified as partial responders (OS: 39 and 15 months; progression-free survival: 14 and 11 months). The ADA results from the JACOB study confirm the low immunogenic potential of pertuzumab, as reported in other indications [1].

Pertuzumab exposure in JACOB was consistent with prior studies in AGC (the phase IIa dose-finding study, JOSHUA, which used 840 mg q3w) and breast cancer (e.g., CLEOPATRA, which used 840 mg loading dose followed by 420 mg q3w). This further supports the existence of a significant difference in the clearance of pertuzumab based on indication. The difference in pertuzumab PK in AGC vs. MBC indications identified in JOSHUA led to the decision to pursue an 840 mg q3w dosing regimen in JACOB rather than the 840 mg/420 mg q3w regimen approved in the HER2-positive breast cancer indication [1, 2]. The observed difference in clearance in AGC compared with other indications is consistent with other mAbs and antibody–drug conjugates such as trastuzumab, bevacizumab, and ado-trastuzumab emtansine [20].

It should be noted that exposures in JACOB were limited to *C*_max_ and *C*_min_ only, due to the sparse PK sampling schedule employed in this large Phase III clinical trial. Within cycle pertuzumab, PK samples collected in the previous gastric cancer study (JOSHUA), pooled with the sparse PK samples collected in the JACOB study, were deemed sufficient to characterize pertuzumab PK in this patient population. Here, we rely on *C*_max_ and *C*_min_ only when comparing across geographies or to prior studies such as JOSHUA, ToGA, and CLEOPATRA. This comparison gives a reasonable idea of exposure similarities or differences. Without extensive sampling and the fact that post-dose and pre-dose PK samples were not collected during the same cycles in JACOB, the onset of steady state was concluded to be Cycle 4 for *C*_max,ss_ and Cycle 5 for *C*_min,ss_.

A popPK model of trastuzumab showed that no single covariate could explain the PK difference between AGC and MBC [21]. Hypotheses to explain possible reasons for faster clearance of HER2-targeting molecules in AGC include both target- and non-target-related mechanisms. HER2-target mechanisms include tumor burden and HER2 shed ECD. Tumor burden was ruled out as a potential mechanism in previously published popPK analyses [14]. Shed HER2 ECD was investigated within the JACOB study, as it was a significant covariate for the clearance of trastuzumab or ado-trastuzumab emtansine in MBC, although in these studies, the magnitude of impact was relatively small and not of clinical relevance [22, 23]. In the JACOB study, baseline shed HER2 ECD levels were comparable to levels seen previously in breast cancer patients [F. Hoffmann-La Roche Ltd. Data on file] and there was no apparent relationship between baseline shed HER2 ECD and steady-state pertuzumab *C*_min_.

One promising non-target hypothesis is potential gastric protein leakage or protein-losing enteropathy (PLE) in patients with AGC. There is no direct clinical evidence for a relationship between PLE and the PK of mAbs; however, it is conceivable that PLE could be a driving factor in faster mAb clearance, given that GC is one of the disease states associated with PLE [24] and patients with PLE often develop hypoalbuminemia [25], which is known to be negatively correlated with mAb clearance [9, 14, 21, 23]. Yang et al. [26] showed a decrease in murine IgG1 mAb area under the curve for 0–14 days from 1368 ± 255 to 594 ± 224 μg/mL/day in a murine model of colitis and PLE, providing preclinical support of this potential hypothesis.

Further studies are needed to identify first-line treatment options to improve patient outcomes in HER2-positive AGC and to better identify patients who might benefit from dual anti-HER2 targeted regimens [11]. Using methods beyond the current process for identifying HER2-positive AGC may compensate for the intratumoral heterogeneity observed in GC, which may have negatively impacted responses to targeted therapies in this and other trials [27, 28]. Furthermore, a comprehensive cross-antibody covariate analysis, as well as potential in vitro experiments and/or clinical studies, are warranted to further understand the clearance difference and to support appropriate dosing regimens of other biologics in this indication.

## Electronic supplementary material

Below is the link to the electronic supplementary material.

Supplementary material 1 (DOCX 58 kb)
