# Supplementary material for: Pharmacokinetic and exposure–response analysis of pertuzumab in patients with HER2-positive metastatic gastric or gastroesophageal junction cancer
Source: Cancer Chemother Pharmacol. 2019 Jun 10;84(3):539–50. doi: 10.1007/s00280-019-03871-w (PMC6682857; doi:10.1007/s00280-019-03871-w)
Supplement: Supplementary file 1 — Supplementary material 1 (DOCX 58 kb) [file 280_2019_3871_MOESM1_ESM.docx]

**Supplementary material**

**Pharmacokinetic and exposure–response analysis of pertuzumab in patients with HER2-positive metastatic gastric or gastroesophageal junction cancer**

Cancer Chemotherapy and Pharmacology

Whitney P. Kirschbrown^1^ **·** Bei Wang^1^ **·** Ihsan Nijem^1^ **·** Atsushi Ohtsu^2^ **·** Paulo M. Hoff^3^ Manish A. Shah^4^ **·** Lin Shen^5^ **·** Yoon-Koo Kang^6^ **·** Maria Alsina^7^ **·** Sandhya Girish^1^ **·** Amit Garg^1^

**Corresponding author:** Amit Garg, PhD

Genentech, Inc., South San Francisco, CA, USA

**Email:** garg.amit@gene.com

**Online Resource 1. Baseline demographics and disease characteristics (intention-to-treat population)**

|  | **Pertuzumab + trastuzumab + chemotherapy**  **n=388** | **Placebo + trastuzumab + chemotherapy**  **n=392** |
| --- | --- | --- |
| **Sex** |  |  |
| Male | 294 (76%) | 323 (82%) |
| Female | 94 (24%) | 69 (18%) |
| **Age, years** |  |  |
| Median (IQR) | 62·0 (54·5–69) | 61·0 (54–68) |
| **Geographic region** |  |  |
| Asia (excluding Japan) | 143 (37%) | 146 (37%) |
| Japan | 40 (10%) | 40 (10%) |
| North America/Western Europe/Australia | 133 (34%) | 133 (34%) |
| South America/Eastern Europe | 72 (19%) | 73 (19%) |
| **Measurability** |  |  |
| Measurable disease | 351 (91%) | 352 (90%) |
| Non-measurable evaluable disease only | 37 (10%) | 40 (10%) |
| **Number of metastatic sites*** |  |  |
| 1–2 | 305 (79%) | 303 (78%) |
| >2 | 83 (21%) | 88 (23%) |
| **Histological subtypes (Lauren classification)** |  |  |
| Diffuse | 18 (5%) | 21 (5%) |
| Intestinal | 353 (91%) | 350 (89%) |
| Other^†^ | 17 (4%) | 21 (5%) |
| **Primary site** |  |  |
| Gastro-oesophageal junction | 110 (28%) | 98 (25%) |
| Stomach | 278 (72%) | 294 (75%) |
| **ECOG performance status*** |  |  |
| 0 | 162 (42%) | 162 (41%) |
| 1 | 226 (58%) | 229 (59%) |
| **HER2 status** |  |  |
| IHC 2+ and ISH-positive | 129 (33%) | 130 (33%) |
| IHC 3+ | 259 (67%) | 262 (67%) |
| **Previous gastrectomy** |  |  |
| Yes | 105 (27%) | 102 (26%) |
| No | 283 (73%) | 290 (74%) |

Data are n (%) unless otherwise specified. ECOG= Eastern Cooperative Oncology Group. IHC=immunohistochemistry. ISH=in-situ hybridisation. *n=391 in the control group. †Mixed or indeterminable.

Reprinted from The Lancet Oncology, 19(10), Tabernero J, Hoff PM, Shen L, Ohtsu A, Shah MA, Cheng K, Song C, Wu H, Eng-Wong J, Kim K, Kang YK. Pertuzumab plus trastuzumab and chemotherapy for HER2-positive metastatic gastric or gastro-oesophageal junction cancer (JACOB): final analysis of a double-blind, randomised, placebo-controlled phase 3 study. 1372–1384 Copyright (2018), with permission from Elsevier.

**Online Resource 2.** Pertuzumab steady-state serum trough concentrations across geographic regions


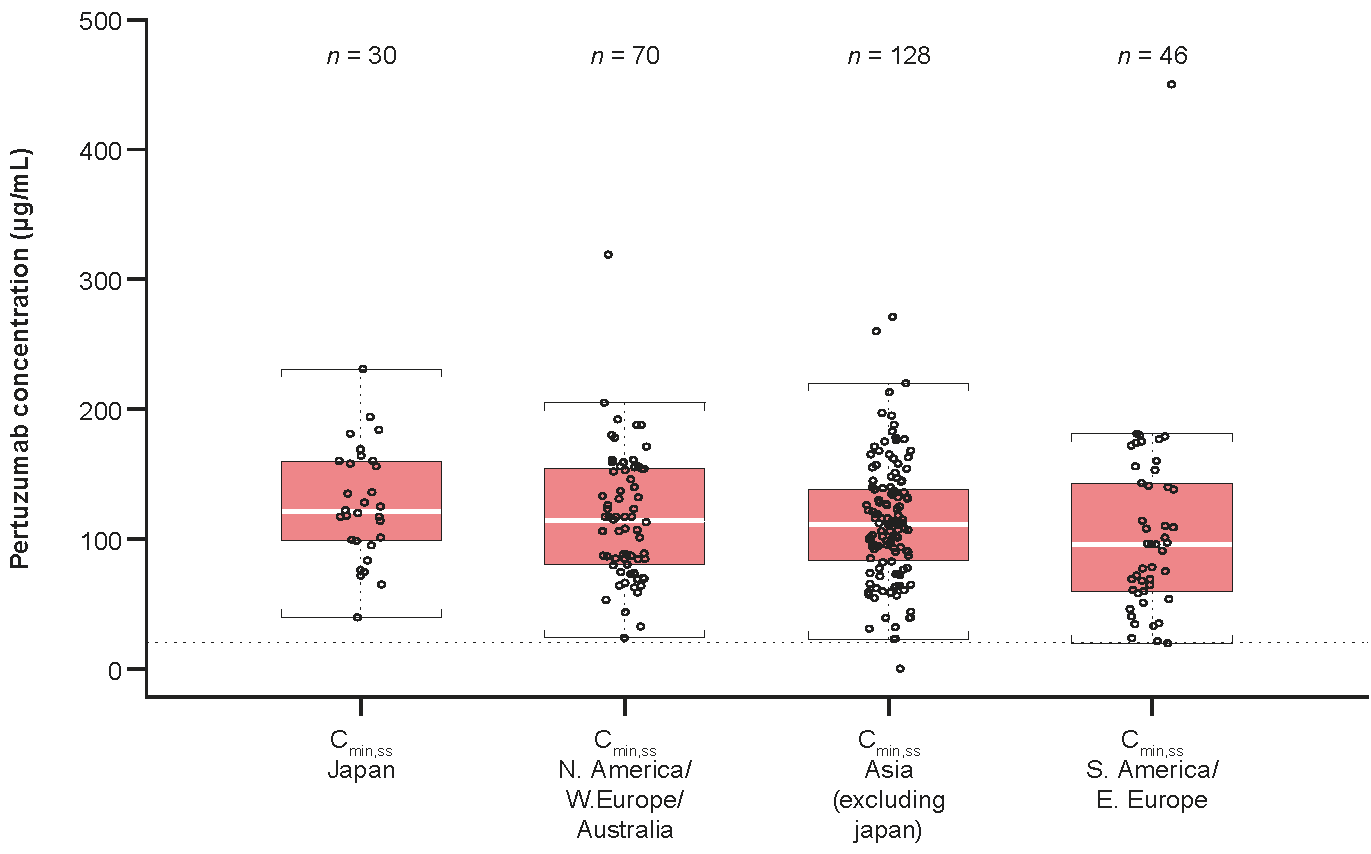


*C_min,ss_* steady-state serum trough (minimum) concentration, *E* eastern, *N* north, *S* south, *W* western.

Lower and upper ends of each box plot = 25^th^ and 75^th^ percentile exposure value; horizontal white line = median per group; points = individual PK data. Brackets extending from the ends of the box are drawn to the nearest value, not beyond 1.5-times the interquartile range

Geographic regions are grouped according to the predefined stratification factors in the JACOB study [11]
